# Alcohol use during the COVID-19 pandemic in Latin America and the Caribbean

**DOI:** 10.26633/RPSP.2021.52

**Published:** 2021-05-20

**Authors:** Rodrigo Garcia-Cerde, Juliana Y. Valente, Ivneet Sohi, Rachael Falade, Zila M. Sanchez, Maristela G. Monteiro

**Affiliations:** 1 Universidade Federal de São Paulo São Paulo Brazil Universidade Federal de São Paulo, São Paulo, Brazil.; 2 Pan American Health Organization Washington DC United States of America Pan American Health Organization, Washington DC, United States of America

**Keywords:** Alcoholism, alcohol drinking, anxiety, quarantine, coronavirus infections, pandemics, mental health, Americas, Alcoholismo, consumo de bebidas alcohólicas, ansiedad, cuarentena, infecciones por coronavirus, pandemias, salud mental, Américas, Alcoolismo, consumo de bebidas alcoólicas, ansiedade, quarentena, infecções por coronavirus, pandemias, saúde mental, América

## Abstract

**Objective.:**

To assess the association between drinking behaviors during the COVID-19 pandemic and quarantine, anxiety symptoms, and sociodemographic characteristics in Latin America and the Caribbean (LAC).

**Method.:**

Data was collected through a cross-sectional online survey (non-probabilistic sample) conducted by the Pan American Health Organization between May 22 and June 30, 2020, in 33 countries and two territories of LAC. Participants were 18 years of age or older and must not have traveled outside of their country since March 15, 2020 (*n*= 12 328, *M* age= 38.1 years, 65% female). Four drinking behaviors (online socializing drinking [OSD], drinking with child present [DCP], drinking before 5 p.m. [DB5]), heavy episodic drinking [HED]) were response variables, and quarantining, anxiety symptoms and sociodemographic covariables were explanatory variables.

**Results.:**

Quarantine was positively associated with a higher frequency of OSD and with DCP, but negatively associated with a higher frequency of HED. Anxiety symptoms were associated with a higher frequency of HED, more OSD, and DB5. Higher incomes seemed to be more associated with all the studied drinking behaviors. Women tended to report less DB5 and less HED during the pandemic.

**Conclusions.:**

Quarantine during the COVID-19 pandemic seems to affect drinking behavior and mental health indicators like anxiety symptoms. This study is the first effort to measure the consequences of the quarantine on alcohol consumption and mental health in LAC during the COVID-19 pandemic. Considering the associations found, screenings and brief interventions targeting alcohol consumption and mental health are recommended.

## RESUMEN

**Objetivo.:**

Evaluar la asociación entre comportamientos relacionados con el consumo de alcohol durante la pandemia de COVID-19 y la cuarentena, los síntomas de ansiedad y las características sociodemográficas en América Latina y el Caribe.

**Método.:**

Se recopilaron datos mediante una encuesta transversal en línea (muestra no probabilística) realizada por la Organización Panamericana de la Salud entre el 22 de mayo y el 30 de junio del 2020 en 33 países y 2 territorios de América Latina y el Caribe. Los participantes tenían 18 años o más y no tenían que haber viajado fuera de su país después del 15 de marzo del 2020 (*n*= 12 328, edad *M*= 38,1 años, 65% mujeres). Las variables de respuesta eran cuatro comportamientos relacionados con el consumo de alcohol: beber socializando en línea, beber en presencia de niños, beber antes de las 5 de la tarde o consumo excesivo episódico. La cuarentena, los síntomas de ansiedad y las covariables sociodemográficas fueron las variables explicativas.

**Resultados.:**

La cuarentena se relacionó positivamente con una mayor frecuencia de consumo de alcohol socializando en línea y en presencia de niños, pero negativamente con una mayor frecuencia de consumo excesivo episódico. Los síntomas de ansiedad se relacionaron con una mayor frecuencia de consumo excesivo de alcohol, un mayor consumo de alcohol socializando en línea y con beber antes de las 5 de la tarde. Aparentemente los ingresos más altos estuvieron más asociados con todos los comportamientos relacionados con el consumo del alcohol estudiados. Las mujeres tendieron a notificar menos consumo de alcohol antes de las 5 de la tarde y menos consumo excesivo episódico de alcohol durante la pandemia.

**Conclusiones.:**

La cuarentena durante la pandemia de COVID-19 parece afectar el comportamiento relacionado con el consumo de alcohol y los indicadores de salud mental, como los síntomas de ansiedad. Este estudio es la primera iniciativa para medir las consecuencias de la cuarentena sobre el comportamiento relacionado con el consumo de alcohol y la salud mental en América Latina y el Caribe durante la pandemia de COVID-19. Dadas las asociaciones encontradas, se recomienda llevar a cabo pruebas de tamizaje e intervenciones breves para abordar el consumo del alcohol y la salud mental.

## RESUMO

**Objetivo.:**

Avaliar a associação entre comportamentos relacionados ao uso de álcool durante a pandemia de COVID-19 e quarentena, sintomas de ansiedade e características sociodemográficas na América Latina e no Caribe (ALC).

**Métodos.:**

Os dados foram coletados em uma pesquisa transversal online (amostra não probabilística) realizada pela Organização Pan-Americana da Saúde (OPAS) em 33 países e dois territórios da ALC entre 22 de maio e 30 de junho de 2020. Participaram da pesquisa pessoas com 18 anos ou mais de idade que não haviam feito viagens ao exterior desde 15 de março de 2020 (*n* = 12 328, mediana [*M*] de idade = 38,1 anos, 65% do sexo feminino). Quatro comportamentos relacionados ao uso de álcool (socialização online com o uso de álcool, uso de álcool na presença de crianças, uso de álcool antes das 5 da tarde e episódios de consumo excessivo de álcool) foram as variáveis de resposta e quarentena, sintomas de ansiedade e covariáveis sociodemográficas foram as variáveis explicativas.

**Resultados.:**

A quarentena teve uma associação positiva com uma maior frequência de socialização online com o uso de álcool e o uso de álcool na presença de crianças, mas demonstrou uma associação negativa com uma maior frequência de episódios de consumo excessivo de álcool. Sintomas de ansiedade foram associados a uma maior frequência de episódios de consumo excessivo de álcool, socialização online com uso de álcool e uso de álcool antes das 5 da tarde. Houve uma aparente associação entre maior nível de renda e todos os comportamentos relacionados ao uso de álcool estudados. As mulheres em geral relataram menos uso de álcool antes das 5 da tarde e menos episódios de consumo excessivo de álcool durante a pandemia.

**Conclusões.:**

A quarentena durante a pandemia de COVID-19 parece influenciar o comportamento relacionado ao uso de álcool e indicadores de saúde mental, como sintomas de ansiedade. Este é o primeiro estudo que procurou dimensionar a repercussão da quarentena no uso de álcool e na saúde mental das pessoas na ALC durante a pandemia de COVID-19. Diante das associações observadas, recomenda-se rastrear problemas relacionados ao uso de álcool e de saúde mental e oferecer intervenções breves.

The novel severe acute respiratory syndrome coronavirus (SARS-CoV-2), known as COVID-19, rapidly spread around the world after first being identified in December 2019 in China (1). By 11 March 2020, COVID-19 was declared a pandemic by the World Health Organization (1). The pandemic resulted in several governments in the Americas to order mandatory closure of all non-essential services and businesses. National quarantines, curfews, and shelter-in-place policies were implemented to decrease the spread of the virus. In some countries, liquor stores were considered essential, while other countries completely banned the sale of alcoholic beverages (2–5).

Alcohol poses many acute and chronic risks to health and is associated with an increased risk of weakening the immune system which can make individuals more susceptible to contracting infectious diseases, including COVID-19 (6,7). Heavy use of alcohol increases the risk of acute respiratory distress syndrome (ARDS), which is one of the most severe complications of COVID-19 (6).

Alcohol consumption shifted from public and licensed premises to the homes of individuals in order to manage the stressful adaptive challenges of COVID-19 (6,8). Higher rates of negative mental distress symptoms like stress, anxiety, fear, and worrying can lead to the commencement, maintenance, or increase of alcohol use (8,9). It is well-documented that alcohol use can increase during difficult time periods like disasters and pandemics and, importantly, it may not automatically normalize post-disaster or pandemic (9–12).

In this context, scientific literature has raised two hypotheses about what would happen in drinking behaviors in the wake of the COVID-19 pandemic, based on literature review of previous health emergencies: they might increase due to stress, or they might reduce due to lack of access to alcohol and changes in social drinking dynamics (3,13).

Online surveys were conducted in Latin American countries like Argentina, Brazil, Colombia, and Mexico (14–16). These surveys covered mental health symptoms but only included one or just a few questions on alcohol consumption. In order to cover more countries and a achieve a more detailed assessment of alcohol consumption and associated risks during the pandemic, the Pan America Health Organization (PAHO) conducted a regional study focused on alcohol consumption before and during the pandemic in all countries of Latin America and the Caribbean (3,17). The descriptive results of this regional study were already published in a special report (18).

Our aim was to assess the association between drinking behaviors during the COVID-19 pandemic period and quarantine, anxiety symptoms, and sociodemographic variables in respondents from Latin America and the Caribbean.

## METHODS

### Study design and setting

**FIGURE 1.**
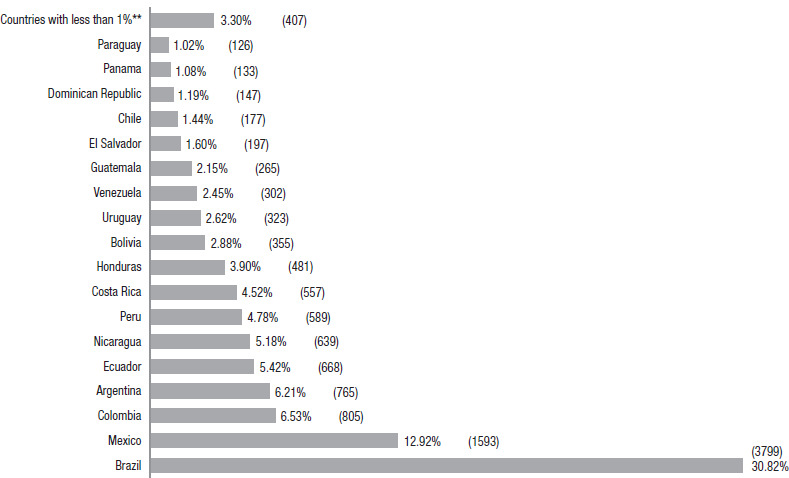
Proportion of respondents by country in the “Alcohol Use during the COVID-19 pandemic in Latin America and the Caribbean” survey, 2020 (*N* = 12 328)^*^

The original study was a cross-sectional, non-probabilistic, PAHO web-based survey (18). It was disseminated through PAHO’s communication platforms —including Facebook, Twitter, the Pan American Network for Alcohol and Public Health (PANNAPH)—, other networks —including the Healthy Caribbean Coalition, the Healthy Coalition of Latin America—, as well as through PAHO staff and direct contacts. The survey covered 33 countries and two territories in Latin America and the Caribbean (Figure 1). Participants answered the online questionnaire, anonymously, from 22 May to 30 June 2020.

* The numbers in parentheses are the corresponding participants in each country.

** Corresponding to countries with less than 1% of the whole sample: Trinidad and Tobago (n=99), Belize (n=97), Suriname (n=35), Barbados (n=32), Guyana (n=30), Grenada (n=24), Curaçao (n=20), Saint Kitts and Nevis (n=16), Saint Lucia (n=10), Haiti (n=9), Antigua and Barbuda (n=7), Bahamas (n=8), Dominica (n=8), Jamaica (n=6), Cuba (n=4), Aruba (n=1), and Saint Vincent and the Grenadines (n=1).

### Participants and sample size

Inclusion criteria was being 18 years old or older, resident of one of the Latin American or Caribbean countries or territories, and not having traveled out of their country since 15 March 2020. The Qualtrics software (Qualtrics, Provo, Utah, United States) was used to register any attempt to participate in the survey; of the 23 058 respondents who accessed the weblink, 12 328 (53.50%) agreed to participate and were considered valid in this analysis. Valid questionnaires were the ones that fulfilled the inclusion criteria and completed answers for the first block of alcohol consumption questions.

### Instrument

A questionnaire standardized in four languages (English, Spanish, Portuguese, and French), consisted of 55 questions that covered sociodemographic characteristics; COVID-19 related questions, including precautionary measures taken in daily life; anxiety symptoms in the past 14 days (19,20); drinking behaviors in 2019; and drinking behaviors during the COVID-19 pandemic period.

### Variables

**Drinking behaviors.** Response variables were four drinking behaviors: online socializing drinking (OSD) (21), drinking with children present (DCP) (22), drinking before 5 p.m. (usually considered as pre-drinking) (DB5) (23), and heavy episodic drinking (HED). HED was defined as consuming at least 5 standard drinks in a single occasion at least monthly. A standard drink was defined as a drink containing approximately 14 grams of pure alcohol (24).

The first three are continuous variables measuring the perceived percentage of times participants consumed alcohol. Questions were formulated as follow: For OSD: “How much (in percentage of times) of your alcohol consumption occurred while socializing with friends or family online?” For DCP: “(…) while there were children under the age of 13 present?” And, for DB5: “How often did you have your first drink of the day before 5 p.m.?”

HED was an ordinal variable measuring the frequency of the behavior. It had seven categories, from “never” to “everyday”. The question was formulated as follows: “How often did you drink five or more standard drinks in one occasion? (One standard drink is equivalent to a can of beer (355 ml), a glass of wine (150 ml), or a shot of distilled spirits (40 ml)”. These four variables were asked for 2019 and the COVID-19 pandemic period in 2020.

**Anxiety symptoms.** The first main explanatory variable was anxiety symptoms measured with the 7-item Generalized Anxiety Disorder Scale (GAD-7) and one question on the quality of sleep, considering the negative consequences in mental health of confinement regarding to sleeping (25). It was decided to use this scale because it has been validated for primary care patients, general population, and adolescents with generalized anxiety disorder in several countries (19,20). Participants were asked about these eight items experienced in the past 14 days. We transformed them dichotomously by assigning the responses “not at all” and “several days”, for the negative category and “over half the days” and “nearly every day” for the affirmative category. However, in our analysis we used a variable that sums the number of anxiety symptoms (including the quality of sleep question) reported by each participant. It had nine categories from “no symptoms” to “eight symptoms”. We treated this variable as numeric.

We used confirmatory factor analysis to provide evidence for the construct validity of the questions about anxiety symptoms used. To evaluate the goodness of fit, we used the comparative fit index (CFI), the Tucker-Lewis index (TLI), and root mean square error approximation (RMSEA). The cutoff criteria used to determine the goodness of fit were an RMSEA estimate near or less than 0.08, and CFI and TLI near or greater than 0.90 (26). Mplus version 8.0 (Mplus: Statistical Analysis with Latent Variables, Los Angeles, California: authors) was used to run this analysis. The fit indices indicated a good model, with X² = 311.028 and p-value < 0.001, RMSEA estimate = 0.034, RMSEA probability = 1.000, CFI = 0.991, and TLI = 0.988. All the factor loadings were greater than 0.7, as required by Hair et al. (27).

**Quarantine.** The second main explanatory variable was quarantine. This variable is dichotomous and was constructed using questions on precautionary measures, based on the WHO prevent measures against the spread of COVID-19 (28). For the affirmative category, we included those who answered one or more of the following statements: working/studying from home, asked to stay in isolation at home after travel overseas, staying in isolation at home, quarantined to a hotel room, and isolated people within home. For the negative category, we included those who responded that they had not taken any precautionary measure and those who only answered “avoiding public transport and social gatherings” or “home-schooling children/keeping pre-school children home from daycare”.

**Sociodemographic characteristics.** Control variables were sex (men and women), age (18 to 81 years), income (from less than one to more than 20 monthly wages) (29), and ethnicity (white, black, indigenous, and mixed/others).

### Statistical analysis

Stata SE version 16 (StataCorp. 2019. Stata Statistical Software: Release 16. College Station, Texas: StataCorp LLC) was used to perform the statistical analysis, consisted of two steps detailed below. As this analysis was not pre-registered, the results should be considered exploratory.

**Step 1: Descriptive analysis.** For descriptive analysis, we calculated the prevalence or means of sociodemographic characteristics, drinking behaviors, anxiety symptoms, and quarantine, both for the whole sample and for drinkers (that is, respondents that have reported drinking in 2019). Comparisons between men and women were performed using t-test or chi-square test according to the variable. Descriptive statistics are presented as percentages and the level of significance for the univariate tests was set at 5%.

**Step 2: Regression analysis.** To answer our objective, four regression models were performed (univariate and multivariate), in which we estimated the average change in drinking behaviors for the whole sample and for 2019 drinkers. However, since the estimates for both samples (both the whole sample and the 2019 drinkers’ sample) were similar, we present only the results for 2019 drinkers.

For the dependent variables of OSD, DCP, and DB5 (which are quantitative continuous variables), we performed linear regression models, with results given as coefficients. For HED (which is a qualitative ordinal variable), an ordered logistic regression model was performed, with results given as odds ratios. Outcomes are given with their 95% confidence intervals and *p*-values. The level of significance was set at 5%.

For linear regression models, normality assumption was evaluated through the Shapiro-Wilk test. In the three linear models performed, it was pointed out the violation of this assumption. However, according to Wooldridge (30), the central limit theorem shows that estimators based on the Ordinary Least Squares (OLS) method satisfy asymptotic normality. Therefore, for sufficiently large samples like ours (*N* = 5 208), they approach normality. That is why the assumption of normality was relaxed, since the coefficients are consistent and asymptotically non-biased, even with such problems.

In all models, in addition to controlling for the independent variables mentioned, we controlled for the respective drinking behavior in 2019. This variable was the same 2020 drinking behavior question (response variable), but asking for the 2019 behavior. Also, an interaction term was tested between anxiety symptoms and quarantine. The hypothesis behind this was that the joint effect of having anxiety symptoms while quarantining could moderate the magnitude of the association between these main explanatory variables and drinking behaviors. Nevertheless, no statistical evidence of this was found, therefore, we decided to keep the main effects models.

**TABLE 1.** Sociodemographic characteristics, anxiety symptoms and quarantine prevalence of the participants of the “Alcohol Use during the COVID-19 pandemic in Latin America and the Caribbean” survey, 2020 (N = 12 328)

	Total N = 12 328 n (%) or mean±SD	2019 drinkers n = 9 554 n (%) or mean±SD	Men ^b^ n = 3 563 n (%) or mean±SD	Women ^b^ n = 6 881 n (%) or mean±SD
Sex ^a^				
Men	3 563 (34.12)	2 977 (36.00)	3 563 (100.0)	
Women	6 881 (65.88)		5 292 (64.00)	6 881 (100.0)
Age	38.14±12.82	37.79±12.58	38.85±13.05	38.13±12.54
18 to 29 years	3 632 (29.46)	2 857 (29.90)	980 (27.50)	1 982 (28.80)
30 to 39 years	3 619 (29.36)	2 919 (30.55)	1 045 (29.33)	2 058 (29.91)
40 to 49 years	2 550 (20.68)	1 934 (20.24)	742 (20.83)	1 476 (21.45)
50 to 59 years	1 643 (13.33)	1 200 (12.56)	514 (14.43)	902 (13.11)
>60 years	884 (7.17)	644 (6.74)	282 (7.91)	463 (6.73)
Income				
<1 wage	1 684 (16.14)	1 113 (13.45)	522 (14.81)	1 123 (16.54)
1 to 4 wages	3 353 (32.13)	2 546 (30.77)	1 098 (31.15)	2 213 (32.59)
5 to 10 wages	2 388 (22.88)	2 028 (24.51)	818 (23.21)	1 556 (22.91)
11 to 20 wages	1 554 (14.89)	1 317 (24.51)	537 (15.23)	1 004 (14.78)
>20 wages	1 457 (13.96)	1 270 (15.35)	550 (15.60)	895 (13.18)
Ethnicity				
White	4 402 (41.73)	3 598 (43.07)	1 354 (38.07)	3 016 (43.90)
Black	443 (4.20)	318 (3.81)	140 (3.94)	299 (4.35)
Indigenous	154 (1.46)	107 (1.28)	56 (1.57)	92 (1.34)
Mixed & others	5 551 (52.62)	4 331 (51.84)	2 007 (56.42)	3 463 (50.41)
Anxiety symptoms				
Feeling nervous, anxious or on edge	3 471 (28.16)	2 785 (29.15)	809 (22.71)	2 079 (30.21)
Not being able to stop or control worrying	2 427 (19.69)	1 894 (19.82)	563 (15.80)	1 462 (21.25)
Worrying too much about different things	2 773 (22.49)	2 202 (23.05)	648 (18.19)	1 650 (23.98)
Trouble relaxing	3 242 (26.30)	2 600 (27.21)	775 (21.75)	1 957 (28.44)
Being so restless that it is hard to sit still	2 182 (17.70)	1 728 (18.09)	555 (15.58)	1 221 (17.74)
Becoming easily annoyed or irritable	2 733 (22.17)	2 205 (23.08)	623 (17.49)	1 636 (23.78)
Feeling afraid as if something awful might happen	2 508 (20.34)	1 984 (20.77)	594 (16.67)	1 429 (20.77)
Having trouble falling sleep	3 280 (26.61)	2 660 (27.84)	828 (23.24)	1 919 (27.89)
Number of anxiety symptoms	1.83±2.43	1.89±2.43	1.51±2.27	1.94±2.47
No one	5 819 (47.20)	4 343 (45.46)	1 914 (53.72)	3 103 (45.10)
One	1 827 (14.82)	1 458 (15.26)	535 (15.02)	1 019 (14.81)
Two	1 115 (9.04)	904 (9.46)	280 (7.86)	651 (9.46)
Three	836 (6.78)	663 (6.94)	199 (5.59)	488 (7.09)
Four	665 (5.39)	548 (5.74)	177 (4.97)	374 (5.44)
Five	573 (4.65)	456 (4.77)	118 (3.31)	348 (5.06)
Six	471 (3.82)	373 (3.90)	95 (2.67)	292 (4.24)
Seven	476 (3.86)	379 (3.97)	125 (3.51)	268 (3.89)
Eight	546 (4.43)	430 (4.50)	120 (3.37)	338 (4.91)
Quarantine	10 604 (86.35)	8 406 (88.29)	3 026 (85.22)	6 108 (89.04)

a There were 1 884 missing values in sex variable.

b All comparisons between men and women (chi-square test or t-test) were statistically significant.

### Ethics

This study was conducted in accordance with the Declaration of Helsinki and was reviewed and approved by the Ethics Review Committee of PAHO (number: PAHOERC 0214.01). The participants were informed of the purpose of the study and provided electronic consent prior to completing the questionnaire. Participants could withdraw from the survey at any moment without providing any justification.

### Results

#### Step 1: Descriptive analysis

Figure 1 presents the proportion of respondents by country (countries = 35; *N* = 12 328). The countries with the most respondents were Brazil (30.8%) and Mexico (12.9%).

Table 1 showcases the sociodemographic characteristics, anxiety symptoms, and quarantine prevalence of the participants, stratified by 2019 drinking status and sex. The proportion of men (34.1%) was considerably less than women (65.9%). The same proportions were observed among 2019 drinkers. It was a relatively young sample with an average age of 38.14 years and similar age groups for both sexes. A higher proportion of participants were located in the income level of 1 to 4 monthly salaries. More than half of the sample identified as mixed ethnicity.

**TABLE 2.** Drinking behaviors in 2019 and during the COVID-19 pandemic of the participants of the “Alcohol Use during the COVID-19 pandemic in Latin America and the Caribbean” survey, 2020 (*N* = 12 328)

	Total N = 12 328 n (%) or mean±SD	2019 drinkers n = 9 554 n (%) or mean±SD	Men ^b^ n = 3 563 n (%) or mean±SD	Women ^b^ n = 6 881 n (%) or mean±SD
2019 drinkers	9 554 (77.50)	9 554 (100.0)	2 977 (83.55)	5 292 (76.91)
COVID-19 pandemic drinkers ^a^	7 438 (64.98)	7 296 (82.21)	2 566 (72.02)	4 350 (63.22)
Online socializing drinking				
% frequency - 2019	6.31±16.68	7.64±18.22	7.42±18.58	4.96±14.60
% frequency - COVID-19 pandemic	10.40±22.56	12.82±24.44	11.52±23.62	9.84±21.97
Drinking with children present				
% frequency - 2019	9.21±20.12	11.56±22.05	10.06±20.64	9.17±20.35
% frequency - COVID-19 pandemic	8.07±22.38	9.97±24.60	8.77±22.76	7.80±22.39
Drinking before 5 p.m.				
% frequency - 2019	11.16±20.59	14.04±22.27	14.84±23.69	9.33±18.25
% frequency - COVID-19 pandemic	11.86±23.54	14.76±25.51	15.14±26.54	10.15±21.50
Heavy episodic drinking				
Frequency - 2019				
Never	6 188 (50.19)	3 568 (37.35)	1 328 (37.27)	3 889 (56.52)
Once a month	3 325 (26.97)	3 205 (33.55)	1 090 (30.59)	1 781 (25.88)
Twice a month	1 206 (9.78)	1 188 (12.43)	436 (12.24)	578 (8.40)
3 times a month	379 (3.07)	377 (3.95)	145 (4.07)	170 (2.47)
4 times a month	575 (4.66)	569 (5.96)	247 (6.93)	243 (3.53)
5 to 12 times a month	561 (4.55)	554 (5.80)	267 (7.49)	206 (2.99)
More than 12 times a month	94 (0.76)	93 (0.97)	50 (1.40)	14 (0.20)
Frequency - COVID-19 pandemic				
Never	7 256 (68.02)	5 068 (60.19)	2 039 (57.23)	5 054 (73.45)
Once a month	1 521 (14.26)	1 491 (17.71)	628 (17.63)	865 (12.57)
Twice a month	566 (5.31)	560 (6.65)	268 (7.52)	287 (4.17)
3 times a month	204 (1.91)	201 (2.39)	86 (2.41)	116 (1.69)
4 times a month	480 (4.50)	473 (5.62)	213 (5.98)	256 (3.72)
5 to 12 times a month	523 (4.90)	513 (6.09)	258 (7.24)	262 (3.81)
More than 12 times a month	118 (1.11)	114 (1.35)	71 (1.99)	41 (0.60)

a There were 882 missing values in COVID-19 pandemic drinkers variable.

b All comparisons between men and women (chi-square test or t-test) were statistically significant.

In terms of these demographic variables, our sample was overall consistent with the sociodemographic data for Latin America and the Caribbean (LAC) reported in 2016 by PAHO. It was estimated that 641 millions of people lived in LAC, out of which 21% lived in Brazil and 13% in Mexico. There was a similar proportion between women and men. It was a young population, with the median age in 30 years. The average number of years of schooling was 8.1 (29).

All of the anxiety symptoms were more prevalent in women. The symptom with the highest prevalence was “feeling nervous, anxious or on edge”, and the one with the lowest prevalence was “being so restless that it is hard to sit still”. The average number of anxiety symptoms was higher in women. Most of the sample reported strategies of quarantine (86.3%), but more women in comparison to men. In the univariate analysis we found statistical differences between men and women in all variables.

Table 2 presents descriptive statistics of drinking behaviors variables. In the whole sample, the prevalence of drinking in 2019 was 77.5%, and during the COVID-19 pandemic it decreased to 65%. The average percentage of times for OSD and DB5 increased from 6.31 to 10.40, and from 11.16 to 11.86, respectively. DCP decreased from 9.21 to 8.07. HED decreased in all categories, except in the last ones of higher frequency, in which a slight increase was observed. Statistical differences between men and women were found in all variables in the univariate testes.

#### Step 2: Regression analysis

Tables 3 and 4 present the univariate and multivariate regression models results assessing the association between each drinking behavior and all independent variables, for 2019 drinkers, respectively. In this section, we only report the adjusted results.

Variables positively associated with “OSD during the COVID-19 pandemic” were anxiety symptoms, quarantine, and higher income. Variables negatively associated were to be older in age, and of an ethnicity other than white.

In the model corresponding to “DCP during the COVID-19 pandemic”, quarantine and income were positively associated, and to be older in age was negatively associated.

“DB5 during the COVID-19 pandemic” was positively associated with anxiety symptoms and higher income. To be a woman and of an indigenous ethnicity were negatively associated.

Anxiety symptoms and a higher income were positively associated with “HED during the COVID-19 pandemic”, while quarantine, to be a woman and of an indigenous and mixed ethnicity were negatively associated.

**TABLE 3.** Univariate regression models evaluating the association between drinking behaviors during the COVID-19 pandemic and anxiety symptoms, quarantine and sociodemographic characteristics in 2019 drinkers of the “Alcohol Use during the COVID-19 pandemic in Latin America and the Caribbean” survey, 2020 (*N* = 12 328)

	Online socializing drinking during the COVID-19 pandemic*			Drinking with children present during the COVID-19 pandemic*			Drinking before 5 p.m. during the COVID-19 pandemic*			Heavy episodic drinking during the COVID-19 pandemic**		
	cCoef.	95%CI	*p*-value	cCoef.	95%CI	*p*-value	cCoef.	95%CI	*p*-value	cOR	95%CI	*p*-value
Anxiety symptoms	**0.77**	[0.55; 0.98]	<0.001	**0.42**	[0.20; 0.63]	<0.001	**1.15**	[0.93; 1.37]	<0.001	**1.08**	[1.07; 1.10]	<0.001
Quarantine	**2.88**	[1.65; 4.12]	<0.001	**3.81**	[2.61; 5.02]	<0.001	**1.50**	[0.21; 2.80]	0.023	**0.83**	[0.75; 0.91]	<0.001
Sex												
Men	1			1			1			1		
Women	-0.97	[-2.06; 0.13]	0.084	-0.30	[-1.42; 0.81]	0.591	**-4.77**	[-5.90; -3.63]	<0.001	**0.50**	[0.46; 0.54]	<0.001
Age	**-0.15**	[-0.19; -0.11]	<0.001	<0.01	[-0.04; 0.04]	0.877	<0.01	[-0.04; 0.04]	0.952	**0.99**	[0.98; 0.99]	<0.001
Income												
<1 wage	1			1			1			1		
1-4 wages	**4.27**	[2.55; 5.99]	<0.001	**3.66**	[1.93; 5.40]	<0.001	**4.70**	[2.92; 6.48]	<0.001	**1.31**	[1.14; 1.51]	<0.001
5-10 wages	**5.55**	[3.76; 7.33]	<0.001	**6.32**	[4.52; 8.12]	<0.001	**7.49**	[5.64; 9.34]	<0.001	**1.27**	[1.09; 1.47]	0.002
11-20 wages	**7.48**	[5.54; 9.43]	<0.001	**6.55**	[4.58; 8.51]	<0.001	**7.65**	[5.63; 9.67]	<0.001	**1.41**	[1.20; 1.65]	<0.001
>20 wages	**7.90**	[5.93; 9.86]	<0.001	**9.38**	[7.40; 11.37]	<0.001	**9.95**	[7.91; 11.98]	<0.001	**1.35**	[1.15; 1.58]	<0.001
Ethnicity												
White	1			1			1			1		
Black	**-3.54**	[-6.35; -0.74]	0.013	**-3.35**	[-6.17; -0.53]	0.020	0.48	[-2.43; 3.39]	0.747	1.07	[0.85; 1.33]	0.566
Indigenous	-3.00	[-7.70; 1.71]	0.212	**-8.88**	[-13.61; -4.14]	<0.001	**-7.02**	[-11.90; -2.14]	0.005	**0.57**	[0.38; 0.85]	0.007
Mixed & others	**-1.69**	[-2.77; -0.61]	0.002	**-4.55**	[-5.64; -3.46]	<0.001	**-2.46**	[-3.58; -1.34]	<0.001	**0.84**	[0.77; 0.91]	<0.001
2019 control variable***	**0.43**	[0.41; 0.46]	<0.001	**0.79**	[0.77; 0.80]	<0.001	**0.72**	[0.70; 0.74]	<0.001	**2.73**	[2.63; 2.83]	<0.001

Abbreviations: “cCoef.”: crude coefficient; “95%CI”: 95% confidence intervals: “cOR”: crude odds ratio.

* Linear regression model.

** Ordered logistic regression model.

*** This control variable is different for each model. It contains the same information as response variable, but corresponds to 2019.

Bold letters highlight statistically significant outcomes.

## DISCUSSION

To our knowledge, our study is the first of its kind to investigate associated factors to drinking behaviors during the COVID-19 pandemic with a large sample from 33 countries and two territories in Latin America and the Caribbean. The sample had a more expressive participation of residents of Brazil and Mexico (these are the most populous of the participating countries), with an overrepresentation of women and young subjects. In summary, quarantine was positively associated with a higher frequency of OSD and with DCP but negatively associated with a higher frequency of HED, both among drinkers and in the total sample. Anxiety symptoms were associated with a higher frequency of HED, more OSD, and DB5. In general, higher incomes seemed to be more associated with all the studied drinking behaviors, contrary to was reported by Wardell et al. in whose study it was observed that the lower the income, the higher the alcohol consumption during the COVID-19 pandemic period (31). Women tended to report less DB5 and less HED drinking during the pandemic. This findings are consistent with Callinan et al., which found a decrease in harmful drinking during social distancing measures in Australia (32).

Our results provide preliminary evidence that social distancing strategies such as stay-at-home orders during the COVID-19 pandemic may have impacted drinking habits, increasing the practice of OSD, and DCP. It is consistent with what was reported by Vanderbruggen et al. and Pollard et al.; these authors observed an increase in alcohol consumption during the COVID-19 pandemic period (33,34). By introducing constraints to social interactions in public spaces and reducing access to public drinking opportunities, an increase in drinking habits within the home such as OSD were observed, and children were more exposed to parental drinking. Previous studies highlighted the negative consequences associated with parental drinking, like increased risk for the early initiation of drinking (35,36) and alcohol-related negative consequences (37). Exposing children more frequently to parental alcohol consumption may endorse lower risk perception of negative consequences (36) and positive expectations surrounding alcohol use (37) and may also promote a more accessible environment that may facilitate their teenage children’s initiation to drinking (38).

At the beginning of the COVID-19 pandemic, it was not clear which direction alcohol consumption would take and two hypotheses were raised based on a literature review of previous public health emergencies: a possible increase due to stress, and a possible reduction due to lack of access (3,13). Findings from the present study point to the negative association between HED and those who reported being in quarantine. One possible explanation for these findings is that working from home, physical distancing rules, and restrictions on social activities may have limited people´s access to alcoholic beverages and reduced their frequency of binge drinking. These results can be explained for by the restrictions of alcohol availability, both financial and physical (3), corroborated from the evidence of alcohol control policy research (39).

Our findings related to anxiety symptoms showed that they were positively associated with patterns of alcohol consumption during the COVID-19 pandemic, such as OSD, DB5, and HED, regardless of the 2019 status of drinking and independent of quarantine. These findings are in line with previous findings that also showed that mental distress was associated with general drinking behavior during the COVID-19 pandemic (31). There is evidence of this association in studies carried out in the United States (40), Australia (41,42), and India (43). These results corroborate previous studies that have documented stress as a prominent risk factor for alcohol misuse (44). However, our study is the first one that we are aware of that showed the effect of mental distress on these specific drinking behaviors: OSD and DB5. It was already documented that an increase in time spent on social media is associated with depression and HED (45). Therefore, we would expect that the stay-at-home order would increase online socializing activities and this could lead to mental distress, and alcohol use can be understood as a way to cope with the negative emotions related to the pandemic (31,46), putting individuals at risk for alcohol-related harms (47). Future research should continue to look at the long-term consequences of the COVID-19 pandemic in relation to mental distress and alcohol use in order to prioritize public mental health actions and to reduce future impacts (48).

**TABLE 4.** Multivariate regression models evaluating the association between drinking behaviors during the COVID-19 pandemic and anxiety symptoms, quarantine and sociodemographic characteristics in 2019 drinkers of the “Alcohol Use during the COVID-19 pandemic in Latin America and the Caribbean” survey, 2020 (*N* = 12 328)

	Online socializing drinking during the COVID-19 pandemic ^a^			Drinking with children present during the COVID-19 pandemic ^a^			Drinking before 5 p.m. during the COVID-19 pandemic ^a^			Heavy episodic drinking during the COVID-19 pandemic ^b^		
	aCoef.	95%CI	*p*-value	aCoef.	95%CI	*p*-value	aCoef.	95%CI	*p*-value	aOR	95%CI	*p*-value
Anxiety symptoms	**0.60**	[0.29; 0.92]	<0.001	0.10	[-0.06; 0.26]	0.204	**0.79**	[0.55; 1.02]	<0.001	**1.04**	[1.03; 1.06]	<0.001
Quarantine	**2.98**	[1.60; 4.36]	<0.001	**1.34**	[0.39; 2.29]	0.007	0.43	[-0.97; 1.83]	0.533	**0.83**	[0.75; 0.92]	<0.001
Sex												
Men	1			1			1			1		
Women	-0.56	[-1.72; 0.60]	0.332	-0.19	[-0.82; 0.44]	0.547	**-1.24**	[-2.31; -0.18]	0.024	**0.73**	[0.67; 0.79]	<0.001
Age	**-0.21**	[-0.27; -0.14]	<0.001	**-0.06**	[-0.09; -0.03]	<0.001	-0.01	[-0.09; 0.07]	0.733	1.00	[1.00; 1.00]	0.963
Income												
<1 wage	1			1			1			1		
1-4 wages	**4.83**	[2.95; 6.71]	<0.001	**1.33**	[0.53; 2.12]	0.002	**2.47**	[0.86; 4.09]	0.004	**1.25**	[1.04; 1.49]	0.015
5-10 wages	**7.16**	[5.15; 9.16]	<0.001	**2.00**	[1.16; 2.84]	<0.001	**5.13**	[3.33; 6.94]	<0.001	**1.44**	[1.26; 1.66]	<0.001
11-20 wages	**10.08**	[7.86; 12.29]	<0.001	**1.86**	[0.41; 3.32]	0.013	**4.66**	[2.67; 6.65]	<0.001	**1.68**	[1.45; 1.95]	<0.001
>20 wages	**10.15**	[8.61; 11.69]	<0.001	**2.64**	[1.13; 4.16]	0.001	**5.91**	[4.47; 7.35]	<0.001	**1.61**	[1.40; 1.85]	<0.001
Ethnicity												
White	1			1			1			1		
Black	**-3.72**	[-6.04; -1.40]	0.003	-0.90	[-3.20; 1.39]	0.431	0.66	[-2.00; 3.32]	0.617	1.07	[0.74; 1.54]	0.735
Indigenous	-2.85	[-6.99; 1.28]	0.170	-1.26	[-3.36; 0.85]	0.233	**-3.50**	[-6.95; -0.05]	0.047	**0.62**	[0.42; 0.92]	0.017
Mixed & others	**-2.22**	[-3.86; -0.57]	0.010	-0.76	[-1.56; 0.05]	0.065	-1.07	[-2.70; 0.56]	0.191	**0.78**	[0.67; 0.92]	0.002
2019 control variable ^c^	**0.43**	[0.36; 0.49]	<0.001	**0.77**	[0.70; 0.85]	<0.001	**0.70**	[0.61; 0.78]	<0.001	**2.69**	[2.55; 2.83]	<0.001

Abbreviations: “aCoef.”: adjusted coefficient; “95%CI”: 95% confidence intervals: “aOR”: adjusted odds ratio.

a Linear regression model.

b Ordered logistic regression model.

c This control variable is different for each model. It contains the same information as response variable, but corresponds to 2019.

All models were estimated with cluster in country. Bold letters highlight statistically significant outcomes.

In our sample, being a man and wealthier were also associated with a higher frequency of each drinking behavior. It is known that in almost all societies (including in all countries of the Americas), men drink more and more often than women (49,50). Previous studies also suggest that the higher the income, the higher the consumption of alcohol (50,51). These factors seem not to be affected by the pandemic.

Considering that quarantine appears to have had an effect on the population´s drinking patterns, independent of several covariates, and the fact that there is no clarity on how it will affect the total population in the long term, more than ever populational interventions are needed to reduce alcohol consumption.

Alcohol consumption is high in the Region of the Americas, and despite efforts made in the last 10 years per capita alcohol consumption has not been significantly reduced (PAHO 2020). It is important not to relax existing alcohol controls aimed at reducing the harmful use of alcohol. This is a prime opportunity to further strengthen the alcohol policies to meet the United Nations’ Sustainable Development Goal of reducing the harmful use of alcohol and per capita consumption by 2030. The population-based policies aimed at reducing the harmful use of alcohol, summarized in the SAFER package created by WHO, can guide actions during the pandemic as well as in the post-pandemic time (50). Providing screening and brief interventions for reducing harmful use of alcohol is an important strategy that can become a part of the rebuilding of health services to become stronger and more responsive to the needs of the people affected by the pandemic (52).

This study has limitations. Firstly, online surveys are not representative of the general population and may have not included those who are heavy drinkers, which limits the generalizability of the results. Secondly, the data is self-reported and subject to memory bias, especially when considering the questions about past behaviors. Thirdly, our cross-sectional design does not allow conclusions about the causality of the associations. Fourthly, for the variables that represent change, the prevalence of events in 2019 considered all 12 months of the year, while for 2020, only 4 months of the pandemic (March to June) were included. Fifthly, because of the on-line survey characteristics, it was not possible to have enough sociodemographic information to perform the analysis of the non-participants. However, given the uniqueness of the questions, the large number of respondents from all countries in Latin America and the Caribbean, the information at subregional and regional levels can be of value to policymakers.

The present study concluded that the quarantine, anxiety symptoms, and sociodemographic characteristics seem to have affected the drinking patterns of the respondents.

## Disclaimer.

Authors hold sole responsibility for the views expressed in the manuscript, which may not necessarily reflect the opinion or policy of the *RPSP/PAJPH* and/or PAHO.
